# Low-Profile Piezoelectric Inertial Linear Actuator for High-Power Applications

**DOI:** 10.3390/mi17040405

**Published:** 2026-03-26

**Authors:** Dalius Mažeika, Regimantas Bareikis, Andrius Čeponis

**Affiliations:** 1 Department of Information Systems, Faculty of Fundamental Sciences, Vilnius Gediminas Technical University, 10223 Vilnius, Lithuania; 2 Department of Mechanical and Materials Engineering, Faculty of Mechanics, Vilnius Gediminas Technical University, 10223 Vilnius, Lithuania; 3 Institute of Mechanical Science, Faculty of Mechanics, Vilnius Gediminas Technical University, 10223 Vilnius, Lithuania

**Keywords:** piezoelectric actuator, linear motion, high preload force, low-profile stator

## Abstract

The paper presents the results of numerical and experimental investigations of a novel low-profile piezoelectric inertial linear actuator designed for a high-payload application. The actuator structure is based on a rectangular piezoelectric bimorph plate with centrally located trapezoidal toothed rings. The actuator operates in the second longitudinal vibration mode of the plate, which is excited by a sawtooth electric signal. Trapezoidal teeth are used to transfer longitudinal vibrations of the plate to the slider and, this way, generate linear motion. The use of trapezoidal teeth reduces the stumbling effect at high preload forces and as a result increases the actuator’s ability to operate under high preload forces and drive higher payloads. Numerical simulations indicated that the actuator exhibits a resonance frequency of 68.49 kHz, with the trapezoidal tooth achieving a maximum displacement amplitude of 188.25 µm at a voltage of 200 V_p-p_. Furthermore, numerical analysis revealed that the trapezoidal tooth deflection in the out-of-plane direction under an axial load of 25 N reached 2.07 nm/N, demonstrating structural stability under high preload conditions. The results of experimental investigations have shown that the actuator can provide up to 75.16 mm/s at a linear motion speed of 200 V_p-p_ and an output force of 18.88 N at the same excitation signal amplitude. In addition, the 15 N load actuator was indicated to achieve a linear motion accuracy of 11.5 µm per step.

## 1. Introduction

High-accuracy and power motion devices such as linear motion actuators and angular motion motors form the backbone of modern mechatronic systems used in advanced optics, robotics, scientific instrumentation, and industrial positioning, orientation, and pointing applications, as well as in space mechatronic and position control systems [1,2,3]. During the last decade, these systems have undergone significant advancements, leading to new challenges in achieving precise control and movement of high payloads with micrometre or nanometre motion resolutions, while maintaining stable operation under dynamically varying payload values and environmental conditions [4,5].

In most cases, these tasks and challenges of modern mechatronic systems are addressed through the use of electromagnetic actuators and motors, as well as their hybrid configuration [6,7]. Although electromagnetic drives offer advantages such as a linear relationship between output force and motion speed, along with a wide dynamic range, they also exhibit several limitations. These include limited scalability, the need for a transmission mechanism, high mass, and a low power-to-volume ratio [8,9]. Moreover, electromagnetic drives induct the self-magnetisation effect, have discrete motion steps, and lack inherent self-locking capability in the absence of power [10]. Consequently, these drawbacks constrain their applicability in driving high-payload systems with high accuracy. In particular, these drawbacks are significant in the development of high-power compact mechatronic systems. Therefore, considering the challenges faced by modern mechatronic systems and the existing limitations of current solutions, replacing conventional electromagnetic drives has become an inevitable decision. Among several possible technologies, piezoelectric actuators and motors are the most promising candidates [11,12,13,14]. Piezoelectric motors and actuators have been under development since the early 60s. The modern form of this type of systems was mostly developed through the work of K.M. Ragulskis and R. Bansevičius and their teams [15,16,17]. Scholars have developed the main principles of piezoelectric motor designs and their operation, which are able to achieve nanometre- to micrometre-scale resolution, self-locking capability without electric power supply, high scalability, magnetic field-free operation, and the ability to drive payloads directly without the need for transmission mechanisms.

However, piezoelectric drives have certain limitations, particularly in terms of output force or torque that depends on the preload force. If the payload exceeds the preload force, slippage and wear occur at the contact interface, leading to a deterioration in performance or eventual failure [18,19,20,21,22]. The preload force can be increased during the motor design stage. For example, in conventional piezoelectric travelling wave motors, this can be achieved by increasing the height of the stator teeth [23]. Nevertheless, the maximum possible height of the tooth is constrained: excessive height causes the tooth to buckle under preload, resulting in inefficient operation, accelerated wear in the contact zone, and potential drive failure [24]. To overcome these limitations, novel structural and design approaches for piezoelectric actuators and motors must be developed to improve load capacity and operational reliability.

Zhang et al. reported on a miniature standing-wave linear ultrasonic motor that operates based on the superposition of the first longitudinal and second bending modes of the stator [25]. The stator comprises a thin stainless-steel beam with two piezoelectric patch plates and two silicon nitride spherical driving tips, which are located at the anti-nodes of the bending mode. The vibrations generated in the stator are transmitted to the slider through these tips, which amplify the displacement amplitude and establish frictional contact between the stator and the slider. Two excitation signals with a 90° phase difference are applied to excite the first-longitudinal and second-bending modes. Such excitation ensures an elliptical motion of the tip that is transferred to the slider via friction force. Numerical and experimental investigations have revealed that the actuator operates at a tuned resonant frequency of approximately 583.5 kHz and exhibits an almost linear relationship between vibration amplitude and excitation voltage. Under an excitation voltage of 20 V_p-p_ and an optimised preload force, the motor achieved a maximum non-load speed of 81.1 mm/s and a driving force of about 56 mN.

Yang et al. designed and investigated a ring-type travelling-wave linear ultrasonic motor [26]. The motor operates on the basis of the third-order in-plane bending vibration of the stator. The stator consists of a circular ring with four sets of incomplete teeth distributed along its outer surface and four piezoelectric ceramic plates positioned at 90° intervals on its inner surface. Two excitation signals with a 90° phase difference are applied to the piezoelectric elements, inducing two third-order orthogonal bending modes that superimpose to generate travelling waves along the stator. The tooth ends on the outer surface amplify these vibrations and transmit them to the slider through friction generated by the slider’s preload force. Numerical investigations have verified the modal shapes of the stator, while experimental measurements have confirmed operation at the resonant frequency of 30.45 kHz under an excitation voltage of 240 V_p-p_. The prototype of the motor achieved a no-load speed of 102 mm/s and a maximum output force of 90 mN.

Ting et al. reported on the short-beam linear travelling-wave piezoelectric motor [27]. The motor used two sets of piezoceramic patches positioned a quarter wavelength apart and driven by two excitation signals with a phase difference of 90°. The superposition of two standing waves in the stator generated a travelling wave along the beam through a series of piezoceramic plates. The stator design incorporated machined gear-shaped teeth, which, together with an alumina contact layer, not only amplify the vibration amplitude but also provide good and wear-resistant surfaces for effective frictional drive against the slider. However, the authors did not investigate the influence of tooth height on the motor’s output force. To suppress wave reflections, a wedge-shaped wave-reduction mechanism was integrated at both ends of the beam. The experimental results demonstrated that the motor reaches a vibration amplitude of approximately 0.38 µm at 45.49 kHz. Under preload, it achieved a maximum output force of 4.8 N, while the maximum speed reached approximately 56 mm/s and a positioning resolution reached up to 280 nm.

Considering the current state of the art, bending modes of vibration of the toothed stators are employed. However, longitudinal vibrations of the stator are more efficient for high-power actuator design. In addition, the geometric characteristics of the teeth have not been investigated; nevertheless, they have a strong effect on the vibrations and the output characteristics of the motor. In this study, a novel low-profile piezoelectric linear actuator with trapezoidal tooth is proposed that operates in the superposition of the longitudinal vibration mode of a plate-type stator and the bending mode of the trapezoidal tooth. The tooth geometry is optimised to promote coupling of resonance-amplified stator vibrations to the slider through the contact interface. This design enables a high output force while maintaining micrometre-scale linear motion resolution.

## 2. Structure and Operation Principle of the Actuator

**Figure 1:**
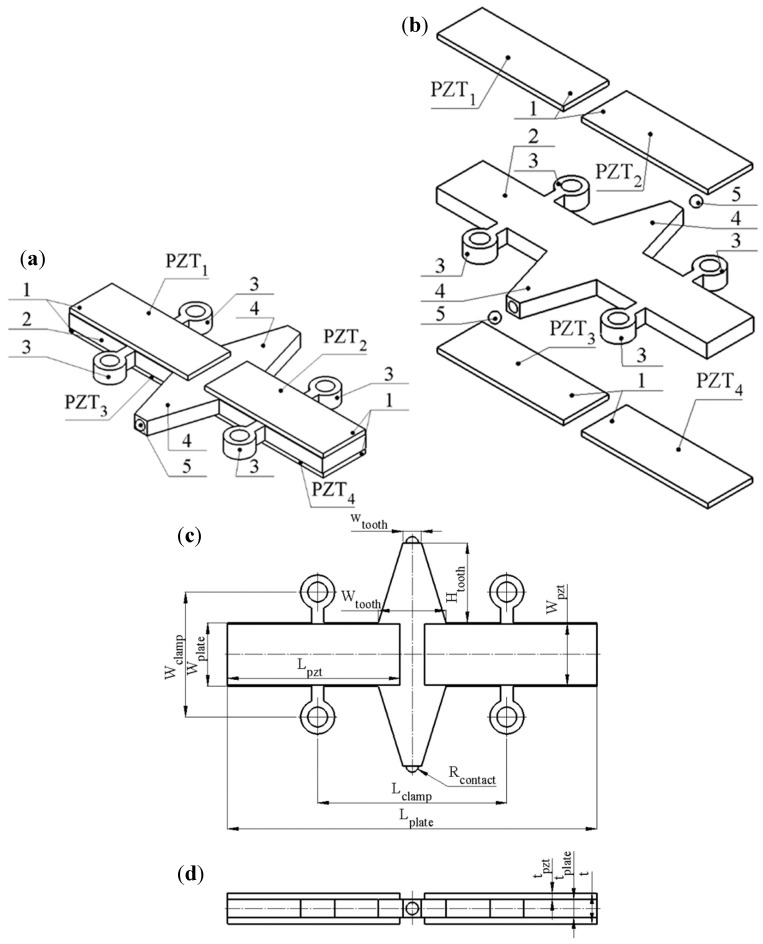
Structure of the stator: (**a**)—assembled view; (**b**)—exploded view; (**c**)—front view; (**d**)—top view; 1—piezoceramic plates; 2—rectangular aluminium plate; 3—clamping beams with holes; 4—trapezoidal teeth; 5—spherical contacts; PZT_1_–PZT_4_—number of piezoceramic plates.

The linear motion actuator is based on a low-profile stator composed of a rectangular aluminium plate. Two trapezoidal-shaped teeth are positioned in the centre of the opposite sides of the plate. A trapezoidal configuration for the tooth was selected to improve the actuator’s load capacity. This geometry enhances buckling resistance and permits the application of higher stator preload forces, and consequently enables greater output force generation compared with rectangular or similar tooth shapes. Four piezoceramic plates, made of a hard piezoelectric material and polarised along the thickness, are bonded to the upper and lower surfaces of the stator. To achieve excitation of the actuator through a single electrical input, the piezoceramic plates are arranged in such a way that the polarisation directions of the piezoceramic plates on the left and right sides are opposite, as are those of the plates on the top and bottom surfaces. Spherical alumina contacts are mounted at the tips of the trapezoidal teeth to increase the contact stiffness between the stator and slider and to ensure efficient transmission of vibrations. The stator is clamped using four clamping beams with the bolt holes for fastening. The structural design, sketch, and geometrical characteristics of the stator are given in Figure 1 and Table 1.

The operation principle of the piezoelectric actuator is based on the superposition of the second longitudinal vibration mode of the rectangular aluminium plate and the first in-plane bending vibrations of the trapezoidal teeth when a non-symmetrical waveform electric signal is applied to the piezoelectric elements. The geometrical parameters of the rectangular plate and the trapezoidal tooth are selected such that the resonant frequencies of the aforementioned vibration modes coincide. The superposition of these modes produces longitudinal vibrations of the spherical contacts, whereas longitudinal vibrations of the piezoceramic plate operate as an excitation source for bending vibrations in the trapezoidal tooth.

The clamping beams are mounted on the rectangular aluminium plate at positions corresponding to the nodal points of the second longitudinal vibration mode. Furthermore, aligning the clamping cylinders with the antinodes of this mode minimises the influence of structural damping, caused by the clamping force, on the operational performance of the stator.

The actuator operates according to the inertial stick–slip principle, induced by a single sawtooth excitation signal whose frequency is close to or equal to the resonance frequency of the second longitudinal vibration mode of the rectangular plate. During the initial stick stage, as the excitation signal gradually increases, the central antinodes move slowly in the forward direction, inducing the bending deformation of the trapezoidal tooth. Meanwhile, the tooth tip and the spherical contact move in the opposite direction.

**Figure 2:**
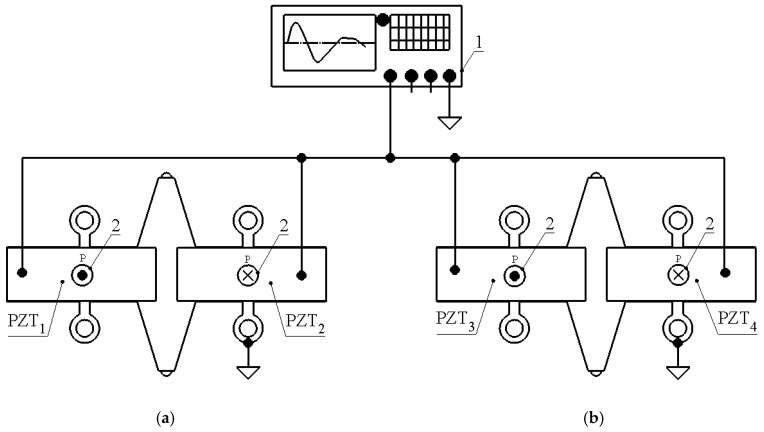
Excitation schematics of actuator; (**a**)—top view of the actuator; (**b**)—bottom view of the actuator; 1—signal generator; 2—polarisation direction; PZT_1_–PZT_4_—number of piezoceramic plates.

During this phase, the friction force at the interface between the spherical contact and the slider is greater than the load force produced by the stator, resulting in a slider displacement consistent with that of the stator tip. When the excitation signal rapidly reverses polarity, the stator changes its motion phase accordingly. The resulting high acceleration and velocity of the spherical contact cause slippage between the contacting surfaces, during which no linear motion of the slider is produced. Through continuous repetition of these stick–slip stages, linear motion of the slider is achieved. To reverse the direction of motion, the phase of the excitation signal must be inverted. A schematic of the stator excitation is presented in Figure 2.

**Figure 3:**
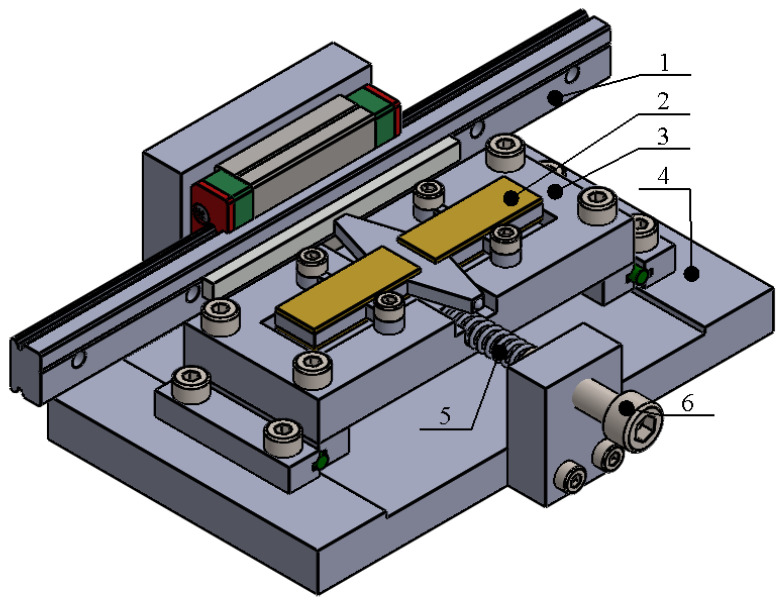
Structural design of the actuator and mounting platform; 1—slider; 2—stator; 3—mounting platform; 4—base; 5—spring; 6—control bolt.

The actuator slider is supported by a linear ball bearing to ensure precise translational motion. To enhance the tribological characteristics of the contact interface between the slider and the stator, the contact surface of the slider is coated with an alumina oxide layer. The stator is mounted onto the platform using clamping cylinders. The platform is guided by linear motion supports, which maintain the stator in a position normal to the slider while permitting controlled displacement along the normal axis. The preload force of the slider is generated by a preload spring that exerts pressure on the platform, thereby pressing the stator against the slider. The preload adjustment is accomplished by adjusting the compression of the spring through the longitudinal displacement of the preload control bolt. The structural design of the actuator is given in Figure 3.

## 3. Numerical Investigation of the Actuator

The actuator was numerically analysed to identify its geometrical and electromechanical characteristics and optimise the dimensions of the trapezoidal tooth. The numerical model was built using the COMSOL Multiphysics 6.1 software. In the initial step, the actuator geometry was defined according to Table 1. The numerical model used the material properties listed in Table 2. The stator was modelled with the aluminium alloy 6061-T6, piezoceramic plates with piezoceramic PIC181 (PI Ceramic GmbH, Lederhose, Germany) and the spherical contacts with alumina oxide. The inner surfaces of the clamping cylinders were fixed and electrical boundary conditions were applied as shown in Figure 2.

Optimisation of the geometric parameters of the trapezoidal tooth was performed in order to achieve the maximum in-plane displacement amplitude of the tip. For this purpose, the optimisation problem was formulated as follows:

(1) $$\begin{array}{c} max\\ H_{tooth},W_{tooth} \end{array}\left(u_{x}\left(H_{tooth},W_{tooth}\right)\right)$$

subjected to

(2) $$H_{tooth}^{min}\leq H_{tooth}\leq H_{tooth}^{max}$$

(3) $$W_{tooth}^{min}\leq W_{tooth}\leq W_{tooth}^{max}$$

where *u_x_* is in-plane displacement amplitude of the tip of the trapezoidal tooth; *H_tooth_* is the height of the trapezoidal tooth; *W_tooth_* is width of the trapezoidal tooth base. $H_{tooth}^{min}\;and\;H_{tooth}^{max}$ are the lowest and highest values of trapezoidal tooth heights, which were set to 11 mm and 13 mm, respectively. $W_{tooth}^{min}\;and\;W_{tooth}^{max}\;$ are the lowest and highest values of trapezoidal tooth base widths, which were set to 11 mm and 13 mm, respectively. The increment step, during calculations, for both parameters was set to 0.5 mm.

**Figure 4:**
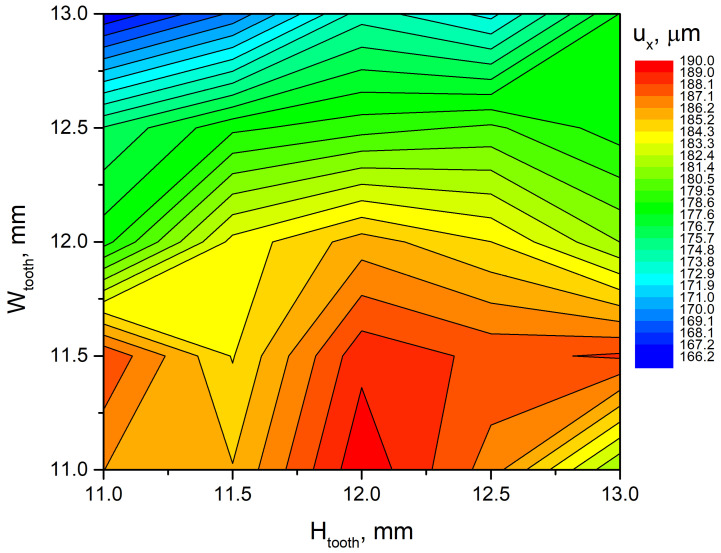
Displacement amplitudes of the tip of the trapezoidal tooth.

The range of *W_tooth_* was selected based on the size of the central antinode region in the second longitudinal vibration mode, ensuring effective interaction between the rectangular plate and trapezoidal tooth, i.e., the base length was limited in order to ensure that the tooth was affected only by this antinode. In turn, the range of *H_tooth_* was defined to cover the natural frequency interval of the tooth, enabling the possibility of achieving superposition with the second longitudinal vibration mode. These ranges therefore represent the physically meaningful domain in which the coupling between structural and longitudinal modes can be optimised. Frequency domain simulations were conducted from 60 to 90 kHz with a 5 Hz resolution and an excitation amplitude of 200 V_p-p_. Spatial increments for both geometric parameters were set to 0.5 mm. The results are presented in Figure 4.

The analysis of the results indicates that the amplitudes of in-plane displacements at the tip of the trapezoidal tooth are directly influenced by its geometric characteristics. The maximum displacement amplitude of 189.77 µm was observed when the tooth width *W_tooth_* and the height *H_tooth_* were 11 mm and 12 mm, respectively. However, the results also reveal that other combinations of geometric parameters can produce similar values of displacement amplitudes. To facilitate a comprehensive assessment, a summary of the combinations of parameters yielding comparable displacement amplitudes is presented in Table 3.

**Figure 5:**
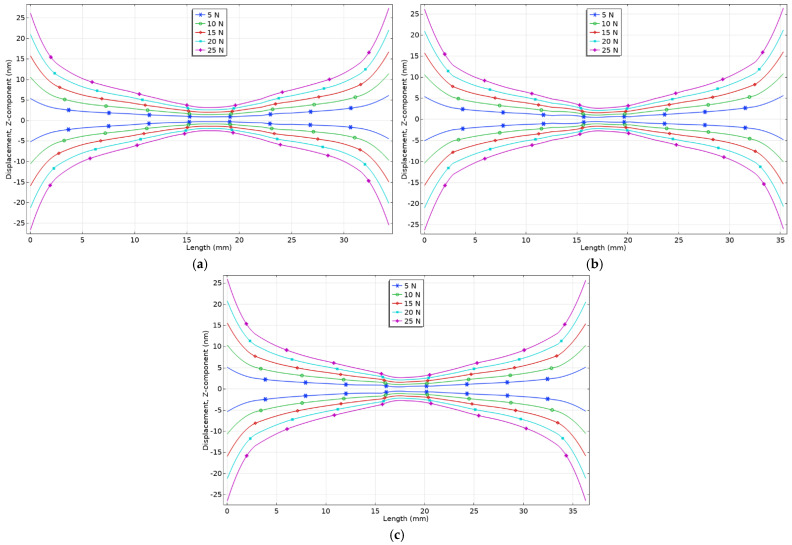
Displacement of trapezoidal tooth under different axial forces: (**a**)—*H_tooth_* = 12 mm, *W_tooth_* = 11 mm; (**b**)—*H_tooth_* = 12.5 mm, *W_tooth_* = 11.5 mm; (**c**)—*H_tooth_* = 13 mm, *W_tooth_* = 11.5.

As shown in Table 3 and Figure 4, the combinations of geometric parameters exhibit nearly identical displacement amplitudes with differences below 1%. To identify the optimal trapezoidal tooth dimensions, numerical analysis of tooth deflections under axial loads was performed. The aim was to determine the configuration minimising out-of-plane displacements when the preload is applied. Axial forces ranging from 5 N to 25 N with an increment of 5 N were applied to the tooth tips, directed toward the stator centre to simulate preload from both sides. The results are presented in Figure 5.

The *x* (horizontal) axis of Figure 5 represents the distance between the tips of the trapezoidal teeth, which are located on both sides of the stator, while the *y* (vertical) axis of the graph represents the displacements of the teeth, while different preload forces are used in the axial direction. Therefore, the analysis of the results reveals that in all three cases, the displacement profiles of the trapezoidal tooth under varying preload forces are symmetric, indicating that the preload induces bending deformations. These deformations are predominantly localised near the tips of the teeth. For the first geometric configuration, the displacement amplitude was 12.2 nm or 2.44 nm/N at a preload of 5 N and 54.78 nm or 2.19 nm/N at 25 N. The second configuration yielded amplitudes of 11.2 nm or 2.24 nm/N and 52.96 nm or 2.11 nm/N, while the third configuration resulted in 10.28 nm or 2.05 nm/N and 51.96 nm or 2.07 nm/N under the same loading conditions. Although variations among configurations are minor, the third configuration demonstrates the lowest displacement amplitudes and, therefore, the highest resistance to preload-induced deflection. Consequently, this configuration was identified as the optimal geometric design of the trapezoidal tooth, providing the highest resistance to preload-induced deformation.

**Figure 6:**
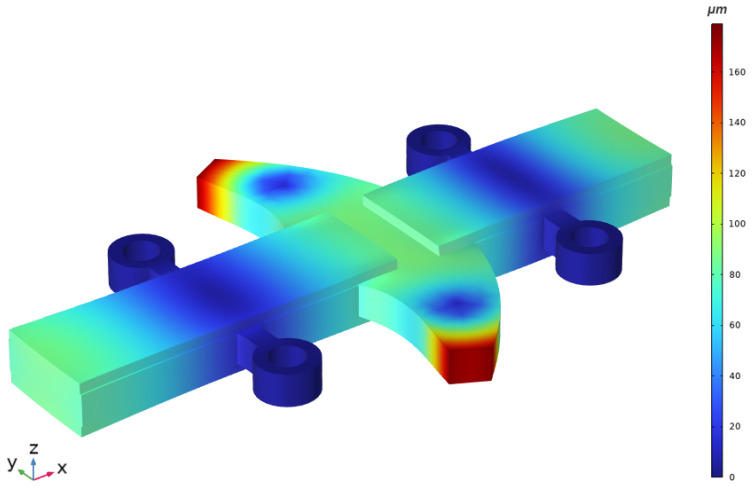
Modal shape of the actuator at 68.49 kHz.

The next phase of the numerical analysis evaluated the modal shapes and natural frequencies of the actuator using the third geometrical configuration of the trapezoidal tooth. The analysis aimed to determine the dominant vibration mode relevant to actuator performance and its corresponding natural frequency. The inner surfaces of the clamping cylinders were rigidly fixed, and an excitation voltage of 200 V_p–p_ was applied. The shape and frequency characteristics of the computed mode is presented in Figure 6.

As shown in Figure 6, the rectangular plate operates in the second longitudinal vibration mode at a frequency of 68.49 kHz. The free-end antinodes of the plate exhibit a 180° phase shift relative to the central antinode, indicating opposite directional motion. In addition, the clamping cylinders coincide with the nodal lines, minimising the structural damping induced by clamping. Both trapezoidal teeth, located at the central antinode, deform in the first bending mode in the plane, indicating the coupling between the second longitudinal mode of the plate and the first bending mode of the tooth. The longitudinal vibrations of the plate induce in-plane bending vibrations of the teeth, enabling linear slider motion via the inertial stick–slip principle when an asymmetrical waveform electrical signal is applied.

**Figure 7:**
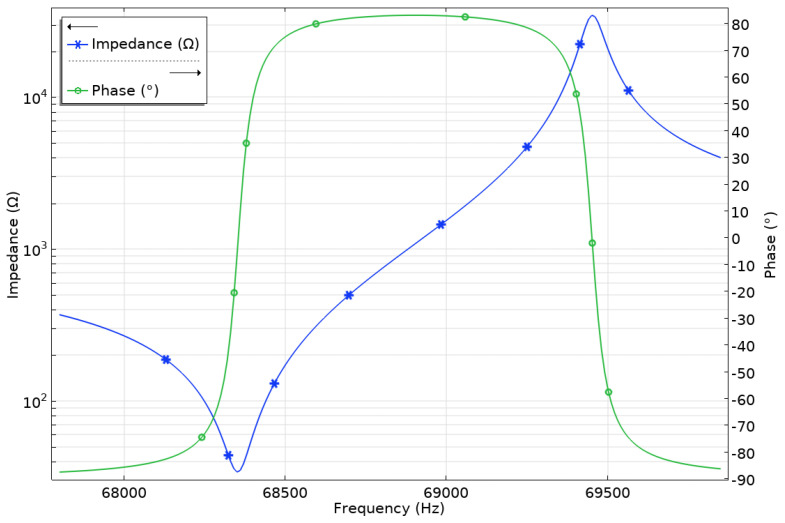
Impedance and phase–frequency characteristics of the actuator.

The next phase of the numerical investigation focused on the impedance and phase analysis in the frequency domain. The frequency range from 67.8 kHz to 68.8 kHz with a calculation step of 5 Hz was investigated. The boundary conditions were identical to those of the previous study. The results are presented in Figure 7.

The resonant frequency of the actuator was determined to be 68.40 kHz, while the anti-resonant frequency was 69.45 kHz. The minor difference of 90 Hz between the natural and resonant frequencies is attributed to the discrete frequency steps used in the impedance and phase–frequency analysis. At resonance, the actuator has an impedance of 34.1 Ω while the phase change is from −87.62° to 82.91°, indicating high-quality resonance and minimal structural damping from the clamping cylinders. The effective coupling coefficient (*k_eff_*) of the actuator was calculated as 0.177, confirming that the actuator operates in the intended vibration modes with efficient energy conversion.

**Figure 8:**
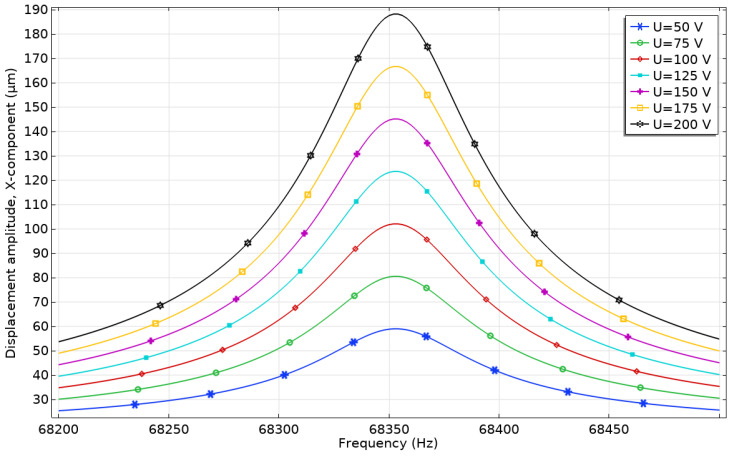
Amplitude—frequency characteristics of the actuator under different voltages.

The displacement of the trapezoidal tooth tip *u_x_* was analysed under varying excitation signal amplitudes (Figure 8). A frequency domain study was performed over 68.2–68.5 kHz with a step increment of 2 Hz, while excitation amplitudes ranged from 50 V_p-p_ to 200 V_p-p_ with an increment step of 25 V_p-p_. The resulting displacement characteristics are shown in Figure 8. Observing the results, it was found that the lowest displacement amplitude of the trapezoidal tooth tip was obtained at the excitation signal amplitude of 50 V_p-p_, reaching 58.98 µm or 1.18 µm/V_p-p_. The highest displacement amplitude of the tip of 188.25 µm or 0.94 µm/V_p-p_ occurred at an excitation amplitude of 200 V_p-p_. Moreover, it can be observed that the change in displacement amplitudes from lowest to highest is 129.27 µm per voltage increase of 150 V_p-p_, or 0.86 µm/V_p-p_. Despite minor variations in the displacement-to-voltage ratio, the actuator demonstrates an approximately linear response to voltage changes. These results indicate that the actuator exhibits reliable and easily controllable displacement characteristics, enabling operation with simple control schemes, either with or without feedback systems.

**Figure 9:**
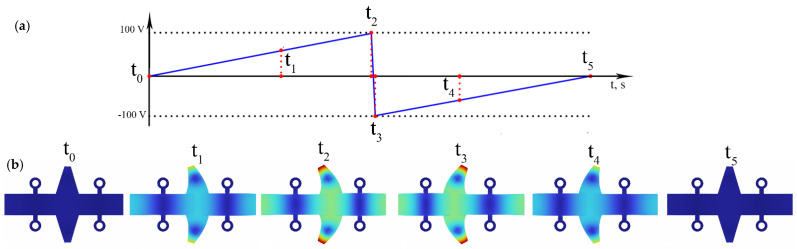
Deformation sequence of the actuator during one period of vibrations; (**a**)—shape of excitation signal; (**b**)—deformation sequence of the actuator.

Finally, a numerical simulation of the actuator’s deformation sequence was conducted to verify its suitability for operation based on the inertial stick–slip principle. A time-domain analysis was performed over one vibration period T, when the saw-tooth waveform excitation signal was applied and the actuator was operated at resonant frequency in a steady-state regime. The calculated time range was 14.63 µs with an increment step of 140 ns. The amplitude of the excitation signal was set at 200 V_p-p_. Figure 9 shows the actuator shapes at six different time points.

When the excitation signal gradually increases from t_0_ to t_2_ or from 0 to 7.32 µs, the rectangular plate and the trapezoidal tooth slowly deform and the stick phase is activated. The frictional force between the stator and the slider is greater than the driving force and the displacement of the slider is generated. When the excitation signal rapidly changes from positive to negative (time range from t_2_ to t_3_ or from 7.32 µs to 7.325 µs), the actuator reverses its displacement phase through the same vibration modes. In this period, the force generated by the stator becomes greater than the frictional force, initiating the slip phase. Subsequently, as the excitation signal varies slowly again between t_3_ to t_5_ or from 7.325 µs to 14.63 µs, the stick phase is reestablished, allowing the displacement of the tip to be transferred to the slider once more. The continuous alternation between these sticks and slip phases produces a constant linear motion of the slider. Reversing the phase of the excitation electric signal results in an inversion of the motion direction.

## 4. Experimental Investigation of the Actuator

**Figure 10:**
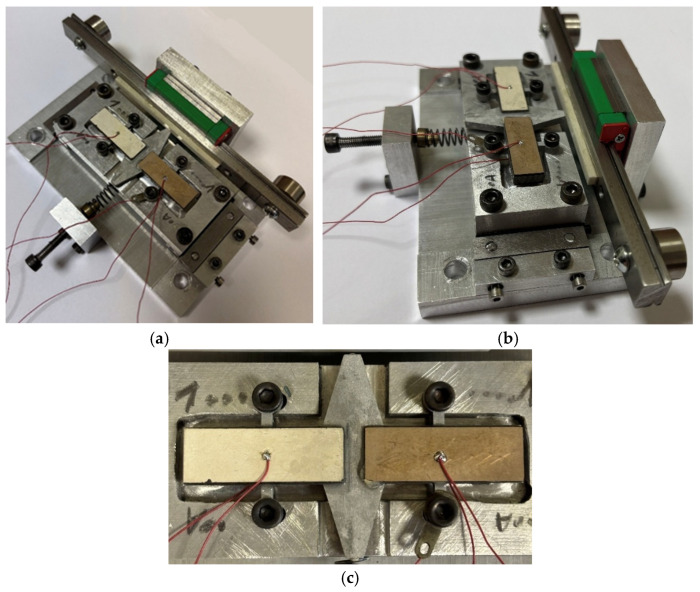
Prototype of the actuator; (**a**)—top view; (**b**)—side view; (**c**)—top view of the stator.

To carry out the experimental investigation, the actuator prototype was made according to the geometric specifications presented in Figure 1 and Table 2. The prototype actuator is shown in Figure 10.

**Figure 11:**
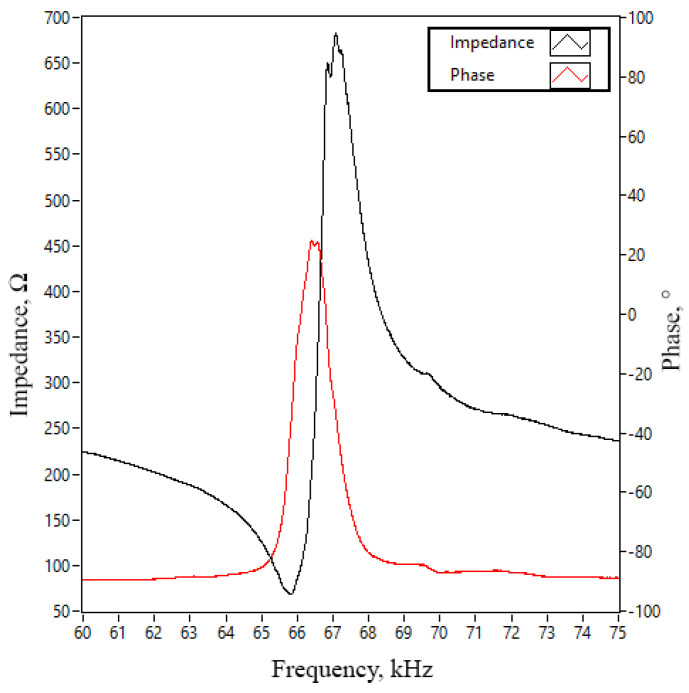
Impedance and phase–frequency characteristics of the actuator in the frequency domain.

Firstly, the impedance and phase–frequency characteristics of the actuator were measured within the frequency range of 60–75 kHz, with a frequency resolution of 2 Hz. For this purpose, a SinPhase 16,667 k impedance analyser was used. Measurements were made in a non-preloaded condition, that is, the tips of the trapezoidal teeth were free, while the stator itself was rigidly clamped to the actuator housing. The obtained results are presented in Figure 11. The resonance frequency of the actuator was observed at frequencies of 65.75 kHz, while the anti-resonance frequency is 67.12 kHz. At resonance frequency, the measured impedance reached 59.32 Ω, while at the anti-resonance frequency it was 669.08 Ω. The effective electromechanical coupling coefficient (*k_eff_*) determined experimentally was 0.193 (Equation (4) [28]). A comparison between the measured and simulated impedance and phase–frequency characteristics reveals certain differences. Specifically, the measured resonance and anti-resonance frequencies are 3.87% and 3.35% lower, respectively, than the corresponding numerically simulated values. Furthermore, the measured impedance at the resonance frequency is 25.22 Ω higher than the calculated value, while the experimentally obtained (*k_eff_*) of the prototype exceeds the simulated value by 9.03%.

(4) $$k_{eff}^{2}=1-{\left(\frac{f_{r}}{f_{a}}\right)}^{2}$$

where *f_r_* and *f_a_* represent resonance frequency and anti-resonance frequencies, respectively.

The differences between the calculated and measured values are induced by manufacturing errors and minor mismatches in material characteristics used to perform numerical calculations and produce the prototype, as well as slight differences in mechanical boundary conditions used in numerical models and for measurements. In the numerical model, the clamping conditions are idealised, whereas the clamping of the prototype does not provide perfectly rigid constraints. The adhesive layer between the piezoceramic plates and the actuator is also neglected in the model, leading to minor discrepancies. In addition, it contains soldered joints and wiring, introducing additional deviations. Despite these differences, the agreement between the numerical and experimental results remains within acceptable limits.

**Figure 12:**
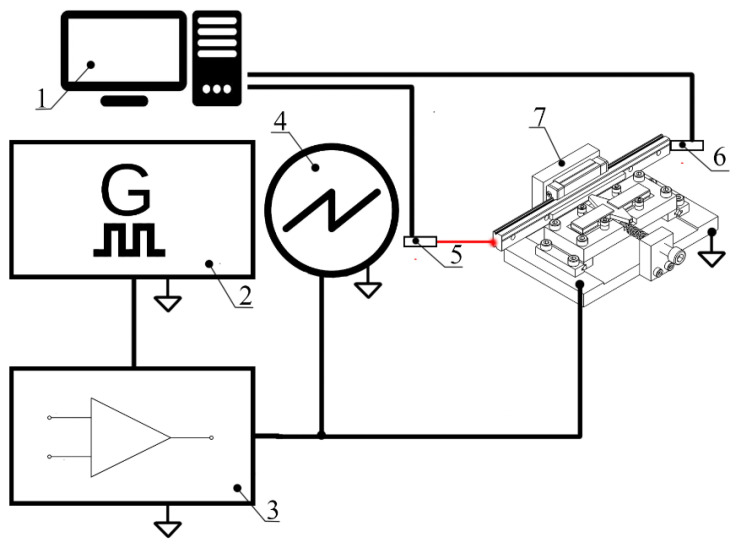
Experimental setup: 1—computer; 2—signal generator; 3—signal amplifier; 4—oscilloscope; 5—non-contact displacement sensor and linear speed measuring device; 6—force sensor; 7—prototype of the actuator.

The subsequent phase of the experimental investigation focused on measuring the mechanical output characteristics of the actuator. Linear motion speed, stall force, and motion accuracy were evaluated under various load and preload conditions. To perform these tests, a dedicated experimental setup was developed, the schematic of which is presented in Figure 12.

**Figure 13:**
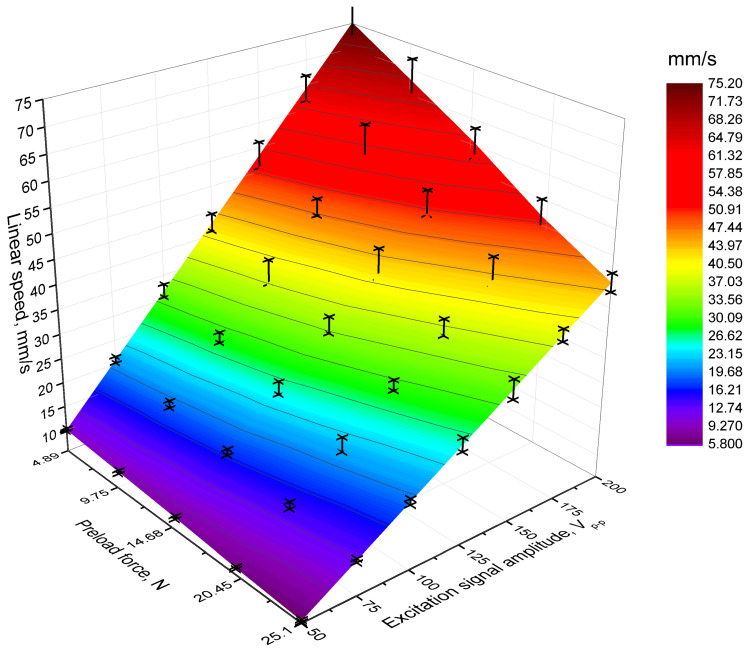
Linear motion speed under different voltages and preload forces.

The experimental setup consists of a computer for data acquisition and processing, a WW5064 function generator generating the sawtooth waveform excitation signal, and a PX-200 power amplifier for signal amplification. The DLM2024 oscilloscope monitored the driving signal amplitude, while the CA1727 non-contact speed sensor and ILD2300 laser displacement sensor measured the slider’s linear speed and displacement. Finally, a PCE DFG N force sensor was used to measure the output force of the actuator. During experimental investigation, excitation signal amplitudes were set to range from 50 V_p-p_ to 200 V_p-p_ with an increment step of 25 V_p-p_, while preload forces were set in the range from 5 N to 25 N with an increment step of 5 N. The results of the measurements are shown in Figure 13.

The analysis of Figure 13 reveals that the lowest linear motion speed of the slider was obtained when the preload force and the amplitude of the excitation signal were set to 25.1 N and 50 V_p-p_, respectively. Under these conditions, the measured linear motion speed reached 5.98 mm/s or 0.119 (mm/s)/V_p-p_. In contrast, the highest linear motion speed was achieved when the amplitude of the excitation signal and the preload force were set to 200 V_p-p_ and 4.89 N, respectively, with a guaranteed linear motion speed of 75.16 mm/s, or 0.375 (mm/s)/V_p-p_. Based on the results, it can be found that the relationship between the linear motion speed, the preload force, and the amplitude of the excitation signal is almost linear and shows stable and predictable behaviour throughout the tested range. Intermediate motion speeds, corresponding to variations in the preload force and excitation amplitude between the lowest and highest tested conditions, exhibit a smooth and consistent trend. This indicates that the motion speed scales proportionally with both parameters, with no significant deviations or nonlinear responses observed.

**Figure 14:**
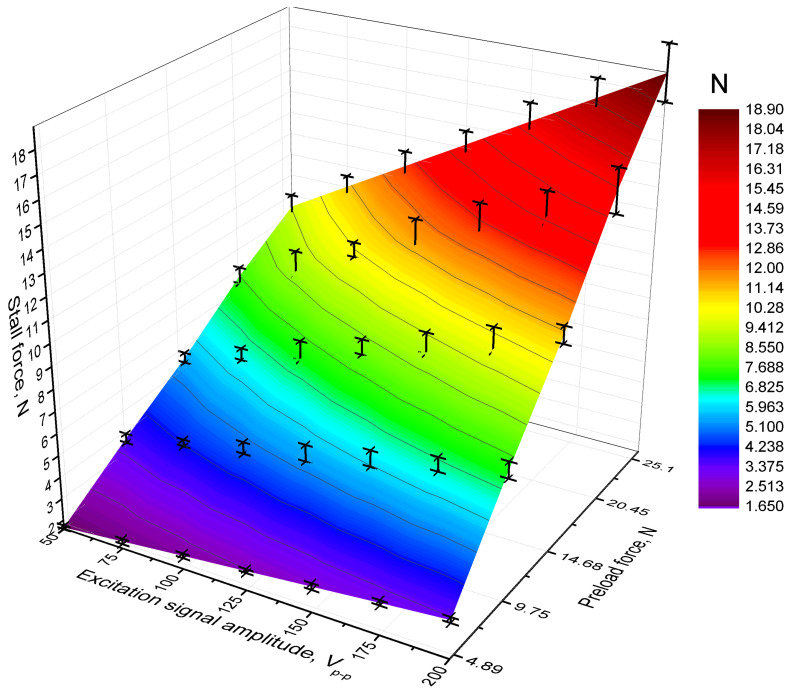
Stall force under different excitation signal amplitudes and preload forces.

The next phase of the experiments was devoted to quantifying the stall force. The measurements were conducted under the same preload forces and excitation voltage amplitudes as in previous tests, with the stall force monitored by a force sensor located at the end of the slider. The resulting data are presented in Figure 14.

The results indicate that the lowest stall force was obtained when the excitation amplitude and preload force were set to50 V_p-p_ and 4.89 N, respectively. Under these conditions, the actuator produced a stall force of 1.66 N or 33.2 mN/V_p-p_. This value represents 33.94% of the applied preload force. On the other hand, the maximum stall force was obtained when the excitation signal amplitude and preload force were set to 200 V_p-p_ and 25.1 N, respectively. At these conditions, the actuator achieved a stall force of 18.88 N, or 94.4 mN/V_p-p_, which is equivalent to 75.21% of the applied preload. The results demonstrate that the stall force increases almost linearly with both parameters, indicating a consistent and predictable relationship between the electrical excitation and mechanical output.

**Figure 15:**
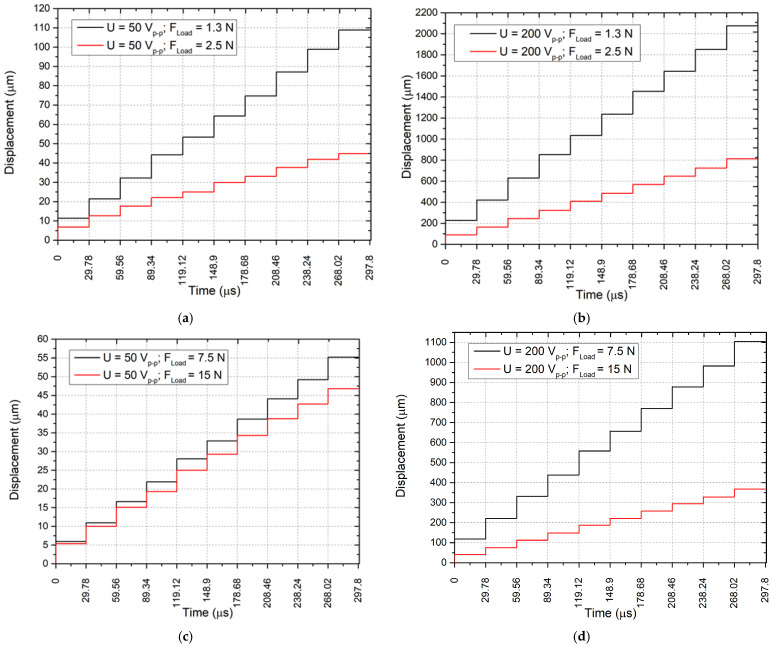
Stepwise mode characteristics of the actuator under loaded conditions; (**a**)—the preload force 4.89 N and excitation signal amplitude 50 V_p-p_; (**b**)—the preload force 4.89 N and excitation signal amplitude 200 V_p-p_; (**c**)—the preload force 25.1 N and excitation signal amplitude 50 V_p-p_; (**d**)—the preload force 25.1 N and excitation signal amplitude 200 V_p-p_.

The next stage of the experimental investigation focused on measuring the linear motion resolution of the actuator under loaded conditions. The excitation signal amplitudes were set at 50 V_p-p_ and 200 V_p-p_ while the preload forces were set at 4.89 N and 25.1 N for both excitation signal cases. The external loads were selected to be approximately 20% lower than the corresponding stall forces, resulting in loads of 1.3 N and 2.5 N for a 4.89 N preload force and 7.5 N and 15 N for a 25.1 N preload force. The actuator was driven by the following conditions: the excitation time for one step was set to around 15.2 µs, which was indicated experimentally as optimal for actuator operation in step mode, i.e., the actuator reaches steady-state vibrations during this time period while a similar time period was set as steady state. So, the total step duration is around 29.78 µs. The displacement of the slider was measured using a displacement sensor, and the results are shown in Figure 15.

The analysis of Figure 15 reveals that at the lower voltage of 50 V_p-p_, the actuator exhibited small and irregular step heights. The smallest step amplitude, approximately 5 µm, was recorded for a preload of 25.1 N and a load of 15 N, indicating that parasitic slippage occurs and influences the step amplitude. These results demonstrate that the low voltage amplitude limits actuator motion due to friction and mechanical resistance, with both preload and load reducing the effective step size nearly linearly. At a voltage amplitude of 200 V_p-p_, a substantial increase in step size was observed, and the motion became smoother and more stable. The highest step amplitude of 215 µm occurred at a preload of 4.89 N and a load of 1.3 N, while the lowest steps of 80 µm were obtained for a preload of 25.1 N and a load of 15 N. The relationship between excitation voltage amplitude and step size is nearly linear. It confirms predictable actuator behaviour, as a higher excitation signal amplitude effectively overcomes frictional resistance and enhances motion stability.

Based on the results obtained, it can be concluded that increasing the preload reduces the step size since a higher normal contact pressure leads to greater frictional resistance and, consequently, to a smaller resulting displacement. However, higher preload conditions also promote more uniform and repeatable step motion, indicating improved contact stability once movement is initiated. At a constant excitation voltage, increasing the external load reduces the amplitude of the step and introduces greater fluctuations between successive steps. This effect is particularly pronounced at low excitation amplitudes, where the actuator has limited driving capability and a larger portion of the vibration energy is consumed to overcome inertial and friction resistance before effective motion is generated. Under such conditions, the motion accuracy reduces because the step-by-step response becomes less consistent and more sensitive to disturbances at the contact interface. From the point of view of motion accuracy, these findings indicate that stable and predictable actuator operation is achieved at higher excitation voltages and at lower-to-moderate preload and load levels. Low-voltage excitation is associated with irregular step formation, reduced displacement resolution, and a higher probability of partial slip, all of which degrade positioning precision. In contrast, higher voltage excitation provides sufficient actuation energy to maintain a stable friction-driven motion regime, resulting in more repeatable step generation and improved controllability. Therefore, the best motion accuracy is achieved when the excitation amplitude is sufficiently high to ensure stable contact-driven motion, while the preload is optimised to balance frictional coupling and displacement output.

## 5. Conclusions

The study introduced and experimentally validated a novel low-profile piezoelectric inertial linear actuator designed for applications requiring both a high preload capability and precise motion control. The actuator consists of a rectangular piezoelectric bimorph stator with trapezoidal teeth positioned at the central antinode of the rectangular plate. The proposed configuration improves mechanical coupling stability and ensures efficient vibration transfer to the actuator slider, enabling reliable operation under high preload conditions. Numerical simulations confirmed that the actuator operates in a coupled longitudinal-bending vibration mode with a resonance frequency of 68.49 kHz and a maximum tip displacement amplitude of 188.25 µm at 200 V_p-p_. The optimised design demonstrated high stiffness and negligible deformation of the trapezoidal teeth under high preloads.

Experimental investigations revealed a maximum linear speed of 75.16 mm/s and a stall force of 18.88 N at 200 V_p-p_. Both output speed and stall force showed an approximately linear dependence on excitation amplitude and preload, indicating stable and predictable actuator performance. Stepwise motion tests further verified the actuator’s capability for precise incremental displacement, with step sizes ranging from 5 µm at 50 V_p-p_ to 215 µm at 200 V_p-p_, while maintaining stable operation under load.

The results obtained indicate that the proposed actuator is promising for compact high-load precision positioning systems, such as optical alignment devices, robotic positioning modules, micro-assembly systems, scientific instruments, and aerospace mechatronic applications, where low-profile, self-locking capability, and magnetic field-free operation are required. However, practical implementation of the actuator will require careful tuning of the preload to specific load conditions, control of contact interface wear, and consideration of environmental influences such as temperature variation, contamination, and mounting stiffness. In addition, high-frequency stick–slip operation may require dedicated drive electronics and control strategies. Therefore, the proposed actuator exhibits strong potential for compact high-precision motion systems.

## Figures and Tables

**Table 1:** Geometrical characteristics of the stator.

Parameter	Value, mm	Description
L_plate_	60.00	Length of rectangular plate
L_clamp_	30.70	Distance between nearby clamping cylinders
L_pzt_	28.00	Length of the piezoceramic plate
W_plate_	10.30	Width of rectangular plate
W_pzt_	10.00	Width of piezoceramic plates
W_clamp_	20.30	Distance between opposite clamping cylinders
W_tooth_	11.50	Width of the base of the trapezoidal tooth
w_tooth_	3.00	Width of the tip of the trapezoidal teeth
H_tooth_	13.00	Height of the trapezoidal tooth
R_contact_	1.00	Radius of spherical contact
t_pzt_	1.00	Thickness of the piezoceramic plate
t_plate_	3.00	Thickness of rectangular plate
t	5.00	Total thickness of the stator

**Table 2:** Properties of the materials.

Material Properties	PI Ceramics PIC181	Aluminium Alloy 6061 T6	Alumina Oxide
Density [kg/m^3^]	7800	2700	3980
Young’s modulus [N/m^2^]	7.6 × 10^10^	6.8 × 10^10^	41.9 ×10^10^
Poisson’s coefficient	0.34	0.33	0.33
Isotropic structural loss factor	-	0.05	0.2 × 10^−3^
Relative permittivity	ε_11_^T^/ε_0_ = 1200 ε_33_^T^/ε_0_ = 1500	-	
Elastic compliance coefficient [10^−12^ m^2^/N]	S_11_^E^ = 11.80 S_33_^E^ = 14.20	-	
Elastic stiffness coefficient c_33_^D^ [N/m^2^]	16.6 × 10^10^	-	
Piezoelectric constant d_33_ [10^−12^ m/V]	265	-	
Piezoelectric constant d_31_ [10^−12^ m/V]	−120	-	
Piezoelectric constant d_15_ [10^−12^ m/V]	475	-	

**Table 3:** Summary of geometrical characteristics with the highest displacement amplitudes.

Combination No.	H_tooth_, mm	W_tooth_, mm	Displacement Amplitude (u_x_), µm	Frequency, kHz
1.	12	11	189.77	69.46 kHz
2.	12.5	11.5	187.83	69.05 kHz
3.	13	11.5	188.25	68.49 kHz

## Data Availability

The original contributions presented in this study are included in the article. Further inquiries can be directed to the corresponding author.
